# Deformed Wing virus absence/presence data across three genera on two Hawaiian Islands

**DOI:** 10.1016/j.dib.2017.12.020

**Published:** 2017-12-15

**Authors:** Jessika Santamaria

**Affiliations:** University of Hawaii, Mānoa, United States

**Keywords:** Deformed Wing virus, pollinator, *Apis mellifera*, *Ceratina smaragdula*, *Polistes*, Hawaii

## Abstract

The data presented in this article relates to the research article, “Evidence of *Varroa*-mediated Deformed Wing virus spillover in Hawaii” (Santamaria et al., 2017) [3]. The article presents data collected throughout August 2014 to November 2015, on the two Hawaiian Islands of Oahu and Maui. *Apis* and non-*Apis* specimens – a total of four species – were collected and tested for Deformed Wing virus (DWV) absence or presence, only. Specific island locations are noted. This data is made publicly available to be analyzed or used in future relevant research.

**Specifications Table**

**Table:** 

Subject area	Entomology, Virology
More specific subject area	Insect virology
Type of data	Table
How data was acquired	A stained SYBR Safe DNA gel stain (Invitrogen) was visualized under ultraviolet light. The positive samples were noted with “1” and negative samples with “0”.
Data format	Raw
Experimental factors	Samples were either from Oahu or Maui
Experimental features	Samples were collected on the two different islands, Oahu or Maui, across different sites. Samples were preserved in −80 °C until they were tested for absence or presence of the Deformed Wing virus.
Data source location	Kahului, Hawaii
	Haleakala, Hawaii
	Iao Needle, Hawaii
	Makawao, Hawaii
	Ho’omaluhia, Hawaii
	Sandy Beach, Hawaii
	Pearl City, Hawaii
	Manoa, Hawaii
	Kaena Point, Hawaii
	Waimanalo, Hawaii
Data accessibility	Data is within this article

**Value of the data**

• Data is useful as benchmark for viral presence in a pre-*Varroa* ecosystem
• Data implicates how important it is to keep *Varroa* out of the *Varroa*-free islands of Hawaii
• Potential future studies can explore pollinator-flower webs and how viruses could spread in these systems

## 1 Data

The data in this article lists which species were collected, from which island, and island location they were collected from. The data shows whether each sample tested positive for Deformed Wing virus (DWV) or not; this is represented by “0” and “1” to indicate absence or presence, respectively. The raw data can be seen in Table 1.

**Table 1:** Four species across three different genera were collected on the Hawaiian Islands of Oahu and/or Maui. Specific island location is noted. Deformed Wing virus (DWV) absence/presence is indicated by “0” or “1” respectively.

DWV	**Species**	**Island**	**Location**
0	*Apis mellifera*	Maui	Kahului
0	*Apis mellifera*	Maui	Kahului
0	*Apis mellifera*	Maui	Kahului
0	*Apis mellifera*	Maui	Kahului
0	*Apis mellifera*	Maui	Kahului
0	*Apis mellifera*	Maui	Kahului
0	*Apis mellifera*	Maui	Kahului
0	*Apis mellifera*	Maui	Kahului
0	*Apis mellifera*	Maui	Kahului
0	*Apis mellifera*	Maui	Kahului
0	*Apis mellifera*	Maui	Kahului
0	*Apis mellifera*	Maui	Kahului
0	*Apis mellifera*	Maui	Kahului
0	*Apis mellifera*	Maui	Kahului
0	*Apis mellifera*	Maui	Haleakala
0	*Apis mellifera*	Maui	Haleakala
0	*Apis mellifera*	Maui	Haleakala
0	*Apis mellifera*	Maui	Haleakala
0	*Apis mellifera*	Maui	Haleakala
0	*Apis mellifera*	Maui	Haleakala
0	*Apis mellifera*	Maui	Iao Needle
0	*Apis mellifera*	Maui	Iao Needle
0	*Apis mellifera*	Maui	Iao Needle
0	*Apis mellifera*	Maui	Iao Needle
0	*Apis mellifera*	Maui	Iao Needle
0	*Apis mellifera*	Maui	Iao Needle
0	*Apis mellifera*	Maui	Iao Needle
1	*Apis mellifera*	Maui	Kahului
1	*Apis mellifera*	Maui	Iao Needle
0	*Ceratina smaragdula*	Maui	Kahului
0	*Ceratina smaragdula*	Maui	Kahului
0	*Ceratina smaragdula*	Maui	Kahului
0	*Ceratina smaragdula*	Maui	Kahului
0	*Ceratina smaragdula*	Maui	Kahului
0	*Ceratina smaragdula*	Maui	Kahului
0	*Ceratina smaragdula*	Maui	Kahului
0	*Ceratina smaragdula*	Maui	Kahului
0	*Ceratina smaragdula*	Maui	Kahului
0	*Ceratina smaragdula*	Maui	Kahului
0	*Ceratina smaragdula*	Maui	Kahului
0	*Ceratina smaragdula*	Maui	Kahului
0	*Ceratina smaragdula*	Maui	Kahului
0	*Ceratina smaragdula*	Maui	Kahului
0	*Ceratina smaragdula*	Maui	Kahului
0	*Ceratina smaragdula*	Maui	Kahului
0	*Ceratina smaragdula*	Maui	Kahului
0	*Ceratina smaragdula*	Maui	Kahului
0	*Ceratina smaragdula*	Maui	Kahului
0	*Ceratina smaragdula*	Maui	Kahului
0	*Ceratina smaragdula*	Maui	Kahului
0	*Ceratina smaragdula*	Maui	Kahului
0	*Ceratina smaragdula*	Maui	Kahului
0	*Ceratina smaragdula*	Maui	Kahului
0	*Polistes exclamans*	Maui	Makawao
0	*Polistes exclamans*	Maui	Makawao
0	*Polistes exclamans*	Maui	Makawao
0	*Polistes exclamans*	Maui	Makawao
0	*Polistes exclamans*	Maui	Makawao
0	*Polistes exclamans*	Maui	Makawao
0	*Polistes exclamans*	Maui	Makawao
0	*Polistes exclamans*	Maui	Makawao
0	*Polistes exclamans*	Maui	Makawao
0	*Polistes exclamans*	Maui	Makawao
0	*Polistes exclamans*	Maui	Makawao
0	*Polistes exclamans*	Maui	Makawao
0	*Polistes exclamans*	Maui	Makawao
0	*Polistes exclamans*	Maui	Makawao
0	*Polistes exclamans*	Maui	Makawao
0	*Polistes exclamans*	Maui	Makawao
0	*Polistes exclamans*	Maui	Makawao
0	*Polistes exclamans*	Maui	Makawao
0	*Apis mellifera*	Oahu	Ho'o Maluhia
0	*Apis mellifera*	Oahu	Ho'o Maluhia
0	*Apis mellifera*	Oahu	Ho'o Maluhia
0	*Apis mellifera*	Oahu	Ho'o Maluhia
0	*Apis mellifera*	Oahu	Sandy Beach
0	*Apis mellifera*	Oahu	Sandy Beach
0	*Apis mellifera*	Oahu	Sandy Beach
0	*Apis mellifera*	Oahu	Sandy Beach
0	*Apis mellifera*	Oahu	Sandy Beach
0	*Apis mellifera*	Oahu	Sandy Beach
1	*Apis mellifera*	Oahu	Ho'o Maluhia
1	*Apis mellifera*	Oahu	Ho'o Maluhia
1	*Apis mellifera*	Oahu	Ho'o Maluhia
1	*Apis mellifera*	Oahu	Ho'o Maluhia
1	*Apis mellifera*	Oahu	Ho'o Maluhia
1	*Apis mellifera*	Oahu	Sandy Beach
1	*Apis mellifera*	Oahu	Sandy Beach
1	*Apis mellifera*	Oahu	Sandy Beach
1	*Apis mellifera*	Oahu	Sandy Beach
1	*Apis mellifera*	Oahu	Sandy Beach
1	*Apis mellifera*	Oahu	Sandy Beach
1	*Apis mellifera*	Oahu	Sandy Beach
1	*Apis mellifera*	Oahu	Sandy Beach
1	*Apis mellifera*	Oahu	Sandy Beach
1	*Apis mellifera*	Oahu	Pearl City
1	*Apis mellifera*	Oahu	Pearl City
1	*Apis mellifera*	Oahu	Pearl City
1	*Apis mellifera*	Oahu	Pearl City
1	*Apis mellifera*	Oahu	Pearl City
1	*Apis mellifera*	Oahu	Manoa
1	*Apis mellifera*	Oahu	Manoa
1	*Apis mellifera*	Oahu	Manoa
1	*Apis mellifera*	Oahu	Manoa
1	*Apis mellifera*	Oahu	Manoa
1	*Apis mellifera*	Oahu	Sandy Beach
1	*Apis mellifera*	Oahu	Pearl City
1	*Apis mellifera*	Oahu	Pearl City
1	*Apis mellifera*	Oahu	Pearl City
1	*Apis mellifera*	Oahu	Pearl City
1	*Apis mellifera*	Oahu	Pearl City
1	*Apis mellifera*	Oahu	Pearl City
1	*Apis mellifera*	Oahu	Pearl City
1	*Apis mellifera*	Oahu	Pearl City
1	*Apis mellifera*	Oahu	Pearl City
1	*Apis mellifera*	Oahu	Pearl City
1	*Apis mellifera*	Oahu	Pearl City
1	*Apis mellifera*	Oahu	Pearl City
1	*Apis mellifera*	Oahu	Pearl City
0	*Ceratina smaragdula*	Oahu	Pearl City
0	*Ceratina smaragdula*	Oahu	Pearl City
0	*Ceratina smaragdula*	Oahu	Pearl City
0	*Ceratina smaragdula*	Oahu	Pearl City
0	*Ceratina smaragdula*	Oahu	Pearl City
0	*Ceratina smaragdula*	Oahu	Pearl City
0	*Ceratina smaragdula*	Oahu	Pearl City
0	*Ceratina smaragdula*	Oahu	Pearl City
0	*Ceratina smaragdula*	Oahu	Ho'o Maluhia
0	*Ceratina smaragdula*	Oahu	Ho'o Maluhia
0	*Ceratina smaragdula*	Oahu	Ho'o Maluhia
0	*Ceratina smaragdula*	Oahu	Ho'o Maluhia
0	*Ceratina smaragdula*	Oahu	Ho'o Maluhia
0	*Ceratina smaragdula*	Oahu	Ho'o Maluhia
0	*Ceratina smaragdula*	Oahu	Ho'o Maluhia
0	*Ceratina smaragdula*	Oahu	Sandy Beach
0	*Ceratina smaragdula*	Oahu	Sandy Beach
0	*Ceratina smaragdula*	Oahu	Sandy Beach
0	*Ceratina smaragdula*	Oahu	Sandy Beach
0	*Ceratina smaragdula*	Oahu	Sandy Beach
0	*Ceratina smaragdula*	Oahu	Sandy Beach
0	*Ceratina smaragdula*	Oahu	Sandy Beach
0	*Ceratina smaragdula*	Oahu	Sandy Beach
0	*Ceratina smaragdula*	Oahu	Manoa
0	*Ceratina smaragdula*	Oahu	Manoa
0	*Ceratina smaragdula*	Oahu	Manoa
0	*Ceratina smaragdula*	Oahu	Manoa
0	*Ceratina smaragdula*	Oahu	Manoa
0	*Ceratina smaragdula*	Oahu	Manoa
0	*Ceratina smaragdula*	Oahu	Manoa
0	*Ceratina smaragdula*	Oahu	Manoa
0	*Ceratina smaragdula*	Oahu	Manoa
0	*Ceratina smaragdula*	Oahu	Manoa
0	*Ceratina smaragdula*	Oahu	Manoa
0	*Ceratina smaragdula*	Oahu	Manoa
0	*Ceratina smaragdula*	Oahu	Manoa
0	*Ceratina smaragdula*	Oahu	Manoa
0	*Ceratina smaragdula*	Oahu	Sandy Beach
0	*Ceratina smaragdula*	Oahu	Sandy Beach
0	*Ceratina smaragdula*	Oahu	Pearl City
0	*Ceratina smaragdula*	Oahu	Pearl City
0	*Ceratina smaragdula*	Oahu	Pearl City
0	*Ceratina smaragdula*	Oahu	Pearl City
0	*Ceratina smaragdula*	Oahu	Pearl City
1	*Ceratina smaragdula*	Oahu	Kaena Point
1	*Ceratina smaragdula*	Oahu	Kaena Point
1	*Ceratina smaragdula*	Oahu	Kaena Point
1	*Ceratina smaragdula*	Oahu	Kaena Point
1	*Ceratina smaragdula*	Oahu	Kaena Point
1	*Ceratina smaragdula*	Oahu	Kaena Point
1	*Ceratina smaragdula*	Oahu	Kaena Point
1	*Ceratina smaragdula*	Oahu	Kaena Point
1	*Ceratina smaragdula*	Oahu	Kaena Point
1	*Ceratina smaragdula*	Oahu	Kaena Point
1	*Ceratina smaragdula*	Oahu	Pearl City
1	*Ceratina smaragdula*	Oahu	Pearl City
1	*Ceratina smaragdula*	Oahu	Pearl City
1	*Ceratina smaragdula*	Oahu	Ho'o Maluhia
1	*Ceratina smaragdula*	Oahu	Ho'o Maluhia
1	*Ceratina smaragdula*	Oahu	Ho'o Maluhia
1	*Ceratina smaragdula*	Oahu	Manoa
0	*Polistes aurifer*	Oahu	Waimanalo
0	*Polistes aurifer*	Oahu	Waimanalo
0	*Polistes aurifer*	Oahu	Waimanalo
0	*Polistes aurifer*	Oahu	Waimanalo
0	*Polistes aurifer*	Oahu	Waimanalo
0	*Polistes aurifer*	Oahu	Waimanalo
0	*Polistes aurifer*	Oahu	Waimanalo
0	*Polistes aurifer*	Oahu	Waimanalo
0	*Polistes aurifer*	Oahu	Waimanalo
0	*Polistes aurifer*	Oahu	Waimanalo
0	*Polistes aurifer*	Oahu	Waimanalo
1	*Polistes aurifer*	Oahu	Waimanalo
1	*Polistes aurifer*	Oahu	Waimanalo
1	*Polistes aurifer*	Oahu	Waimanalo
1	*Polistes aurifer*	Oahu	Waimanalo
1	*Polistes aurifer*	Oahu	Waimanalo
1	*Polistes aurifer*	Oahu	Waimanalo
1	*Polistes aurifer*	Oahu	Waimanalo
1	*Polistes aurifer*	Oahu	Waimanalo
1	*Polistes aurifer*	Oahu	Waimanalo

## 2 Experimental design, materials and methods

### 2.1 Specimen collection

Three species within two different Hymenoptera genera were selected as representatives of the local community of flower visitors: the introduced small carpenter bee *Ceratina smaragdula* (Apidae) which was first recorded in Hawaii in 1999 [2], and the introduced paper wasps *Polistes aurifer* and *Polistes exclamans* (Vespidae) first recorded in Hawaii in the 19th century and in 1952 respectively [1]. *Polistes* wasps collected on Oahu are *P. aurifer* and the specimens from Maui are *P. exclamans*. All samples were collected from either of the five sites on Oahu (*Varroa*-positive island), or the four sites on Maui, (*Varroa*-negative island). Collection sites on both islands consisted on a mix of agricultural fields, parks, gardens, and beach edge vegetation strips. The selected insect species are all relatively abundant and can be found in urban and agricultural environments, where they overlap in resource use with *A. mellifera.*

Samples were collected from August 2014 to November 2015. Insects were collected while they were foraging in fields or flower patches, via a hand-held net. Paper wasp samples were also collected from around their nests. Each insect was stored individually and kept on ice in the field until transferred to a -80 °C freezer for long term storage.

### 2.2 RNA extraction and reverse transcription PCR

Methods for RNA extraction and RT-PCR are fully described in Santamaria et al. [3].
